# The Stroke Alert Project: A Longitudinal Evaluation of a School-Based Stroke Knowledge Intervention

**DOI:** 10.7759/cureus.99828

**Published:** 2025-12-22

**Authors:** Cláudia Santos, Ana R Azevedo, Nádia A Morete, Olívia C Maria, Viorica Gradinaru, Inês Carvalhido, Mariano F Marcos

**Affiliations:** 1 Family Medicine, UCSP Sé, Unidade Local de Saúde do Nordeste, Bragança, PRT; 2 Family Medicine, USF Miguel Torga, Unidade Local de Saúde do Nordeste, Bragança, PRT; 3 Private Practice, UCC Domus, Unidade Local de Saúde do Nordeste, Bragança, PRT; 4 Family Medicine, USF Montesinho, Unidade Local de Saúde do Nordeste, Bragança, PRT

**Keywords:** educational intervention, emergency activation, health literacy, prevention, stroke

## Abstract

Introduction

Stroke is a leading cause of morbidity and mortality in Portugal, and early recognition of symptoms is crucial for timely treatment and improved outcomes. School-based educational programmes may play an important role in increasing stroke-related knowledge among children and, indirectly, their families. This study aimed to evaluate the effect of a school-based educational intervention on stroke-related knowledge among fifth-grade students and to assess knowledge retention immediately after the intervention, one month later, and one year later.

Methods

A quasi-experimental, longitudinal pre-post study was conducted with 149 fifth-grade elementary school students from a public school in Portugal. The educational intervention consisted of a 45-minute interactive session covering stroke definition, recognition of warning signs, appropriate actions in suspected stroke, and primary prevention measures. Stroke-related knowledge was assessed using a 10-item multiple-choice questionnaire administered at four time points: pre-intervention, immediately post-intervention, one month post-intervention, and one year post-intervention. Data analysis was performed using Microsoft Excel® (Microsoft Corp., Redmond, WA, USA). Descriptive statistics, one-way ANOVA with Bonferroni-adjusted post-hoc tests, and independent-samples t-tests were used.

Results

All 149 students completed the four assessments. Mean scores increased from 6.1 ± 1.9 at baseline to 8.7 ± 1.3 immediately after the intervention, 8.1 ± 1.3 at one month, and 7.6 ± 1.8 at one year, corresponding to relative improvements of 42.6%, 32.8%, and 24.5%, respectively, compared with baseline. One-way ANOVA revealed a significant effect of time on knowledge scores (p < 0.001), and all post-intervention scores remained significantly higher than baseline (p < 0.001). No significant differences were observed between male and female students at any time point.

Conclusion

A brief, school-based educational intervention significantly improved stroke-related knowledge among fifth-grade students, with sustained retention over one year, particularly in symptom recognition, appropriate emergency actions and primary prevention measures. Integrating structured, age-appropriate stroke education into school health programmes may be a valuable strategy to strengthen stroke awareness in younger populations.

## Introduction

Stroke remains one of the leading causes of death and disability worldwide, and continues to represent a major public health challenge. It is currently the second leading cause of mortality globally, and a major contributor to disability-adjusted life years (DALYs) lost to non-communicable diseases [1]. It is estimated that one in every four to six individuals will experience a stroke during their lifetime, with approximately 30% dying, and another 30% experiencing permanent disability [2,3]. In Portugal, stroke remains among the main causes of mortality, morbidity, and functional dependence [2-4], accounting for approximately one‑third of all circulatory deaths, and contributing to the highest stroke mortality in Western Europe [5]. Despite this substantial burden, stroke is largely preventable and treatable. Adequate control of modifiable risk factors, such as hypertension, diabetes, dyslipidaemia, smoking, and physical inactivity, could prevent a considerable proportion of cases, while timely access to reperfusion therapies and organised stroke care can markedly reduce mortality and long-term disability [4-6]. However, these benefits depend heavily on early recognition of symptoms and rapid activation of emergency medical services. Delays in seeking help are frequently associated with insufficient knowledge of stroke warning signs or uncertainty regarding the appropriate response when symptoms arise [2,3]. Typical warning signs of stroke include sudden weakness or numbness of the face, arm, or leg (particularly on one side of the body), facial asymmetry, and speech disturbances. Public awareness campaigns and educational initiatives have repeatedly emphasised that “time is brain,” underscoring the importance of promptly recognising these signs and contacting emergency services to enable early treatment and improve outcomes [1-3,7]. Nevertheless, studies consistently demonstrate that knowledge about stroke symptoms and appropriate emergency response remains insufficient in many populations, including in Portugal [3,7-8]. 

Schools represent a strategic setting for health education, particularly in the context of cardiovascular and cerebrovascular diseases. Children and adolescents are highly receptive to structured learning, and educational interventions delivered in school environments may influence not only the students, but also their families and communities [9-12]. Evidence shows that school-based stroke education programmes can significantly improve children’s knowledge of stroke warning signs and risk factors, strengthen their intention to activate emergency services in the event of suspected stroke, and facilitate the transfer of this knowledge to parents and caregivers [13-17]. In this way, children may play an indirect, yet important, role in the stroke emergency response chain. Furthermore, universal school-based health education may contribute to reducing social inequalities in health. As structured and inclusive learning environments, schools provide opportunities to deliver essential health information equitably to students from diverse socioeconomic backgrounds, potentially promoting more uniform levels of awareness among young people [18-21]. 

Within this context, the Projeto Alerta AVC (Stroke Alert Project) was developed as an educational intervention aimed at improving stroke-related knowledge among fifth-grade elementary school students. The programme focused on key domains, including stroke definition, recognition of warning signs, appropriate actions in suspected stroke situations, and primary prevention measures. By targeting a young audience in a structured school setting, the project sought not only to increase awareness but also to explore the persistence of this knowledge over time.

This study aims to evaluate the effect of a school-based educational intervention on stroke-related knowledge among fifth-grade students, and to assess knowledge retention immediately after the intervention, one month later, and one year later. A secondary objective was to analyse differences in knowledge retention by thematic area and gender. 

## Materials and methods

This study followed a quasi-experimental, longitudinal pre-post design to assess changes in stroke-related knowledge among fifth-grade elementary school students at four time points: pre-intervention, immediately after the educational session, one month later, and one year later. The study was conducted between December 2023 and November 2025 by Family Medicine residents, in collaboration with the school health team. 

The study population consisted of fifth-grade students from a public school located within the geographic catchment area of the investigators’ primary healthcare units. A convenience sampling approach was used, as all fifth-grade classes scheduled to participate in the school health programme during the study period were invited. Fifth-grade students were intentionally selected because this age group is routinely included in school health educational activities and demonstrates the developmental capacity to engage effectively with interactive learning. 

Inclusion criteria were attendance at the educational intervention and agreement to complete the questionnaire at each evaluation point. Exclusion criteria included absence from school on the day of the educational session or refusal to participate at any stage of the study. A total of 149 students met the inclusion criteria, and no losses to follow-up occurred. 

The intervention consisted of a 45-minute interactive educational session developed by the investigators in collaboration with the school health team. The session addressed stroke definition, a brief explanation of underlying mechanisms, recognition of warning signs, appropriate actions in suspected stroke situations, and primary prevention measures. Seven sessions were delivered over two days (one per class, 20-22 students per session), using a digital presentation with bright colours, animations, scenario-based questions, and frequent opportunities for student participation. Illustrated handouts summarising key concepts were provided. All content was tailored to the cognitive level and attention span of fifth-grade students. 

A 10-item multiple-choice questionnaire (maximum score of 10 points) was developed by the investigators to assess stroke-related knowledge (see Appendix A). Item content was informed by a review of existing school-based stroke education materials. The questionnaire underwent expert review by Family Medicine specialists to ensure scientific accuracy and by school teachers to assess linguistic clarity and age appropriateness. It was pilot-tested in a group of 20 children of the same age range from another school, resulting in minor adjustments. This process ensured face and content validity. Formal psychometric analyses, such as internal consistency, were not performed, as the questionnaire assessed multiple distinct knowledge domains rather than a single latent construct. 

The questionnaire was administered at four time points: baseline (pre-intervention), immediately after the session, one-month post-intervention, and one-year post-intervention. Only students who attended the intervention completed all follow-up assessments.

**Figure 1:**
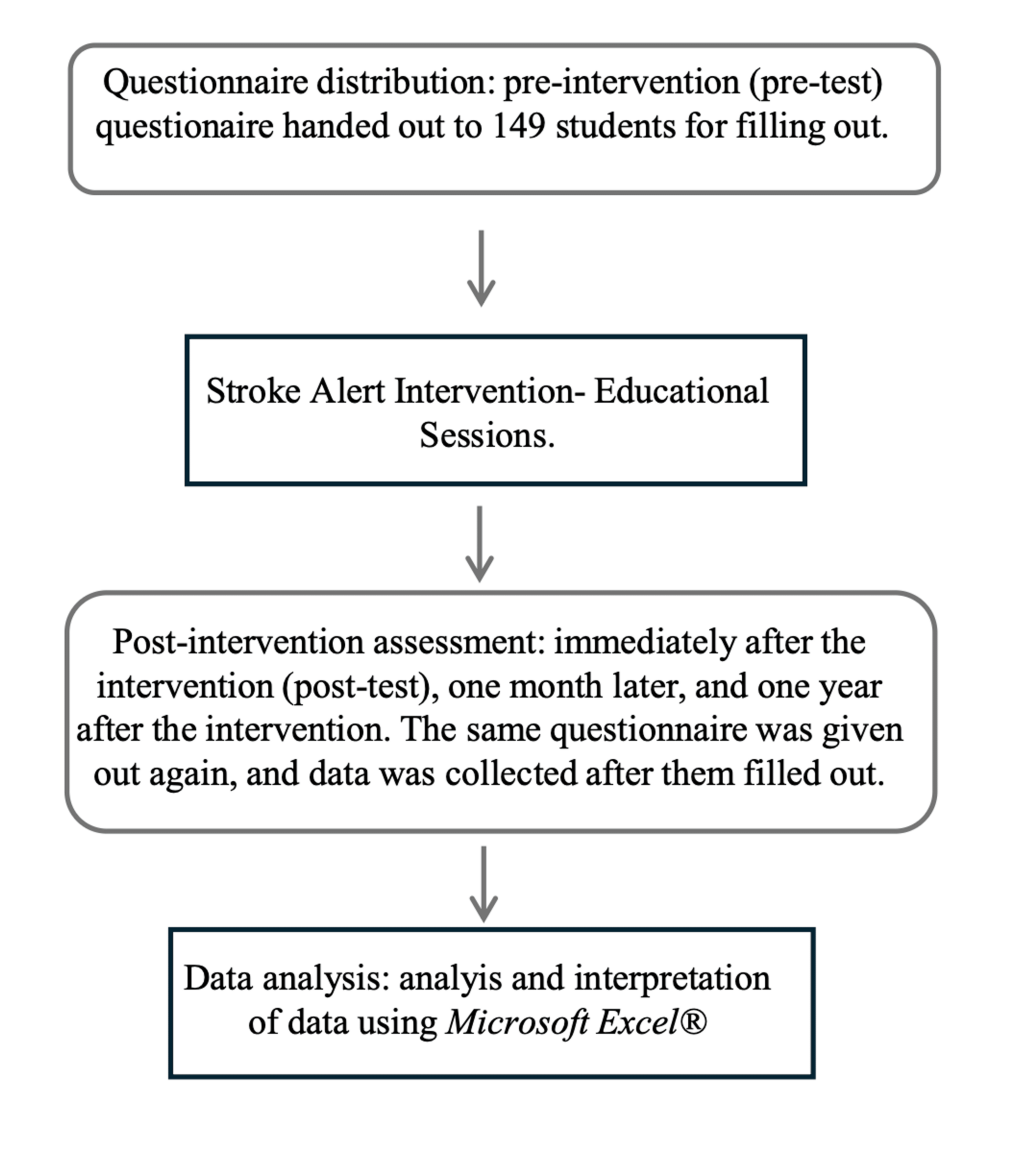
Methods used in the school intervention (Stroke Alert Project)

Data were analysed using Microsoft Excel® (Microsoft Corp., Redmond, WA, USA). Although Excel has limitations for inferential statistics analysis, it supports the standard procedures applied in this study. Descriptive statistics (mean ± standard deviation) were calculated for each time point. A one-way ANOVA, with Bonferroni-adjusted post-hoc tests, was used to assess differences across evaluation periods. Independent-samples t-tests were used to compare male and female students at each time point. Given the large sample size (n = 149), t-tests were considered robust to deviations from normality. As Microsoft Excel® does not support non-parametric alternatives, this limitation was acknowledged as a methodological constraint. The methods used in the school intervention, Stroke Alert Project, are given as a flowchart in Figure 1.

The study was approved by the Ethics Committee of ULS Nordeste (Approval No. 71/2025). The intervention was integrated into the annual school health programme and authorised by the school administration. Parents were informed in writing about the intervention and provided written approval for their child’s participation. Participation was voluntary. Confidentiality and anonymity were ensured throughout the study, in accordance with the Declaration of Helsinki and relevant national guidelines.

## Results

Demographics

**Figure 2:**
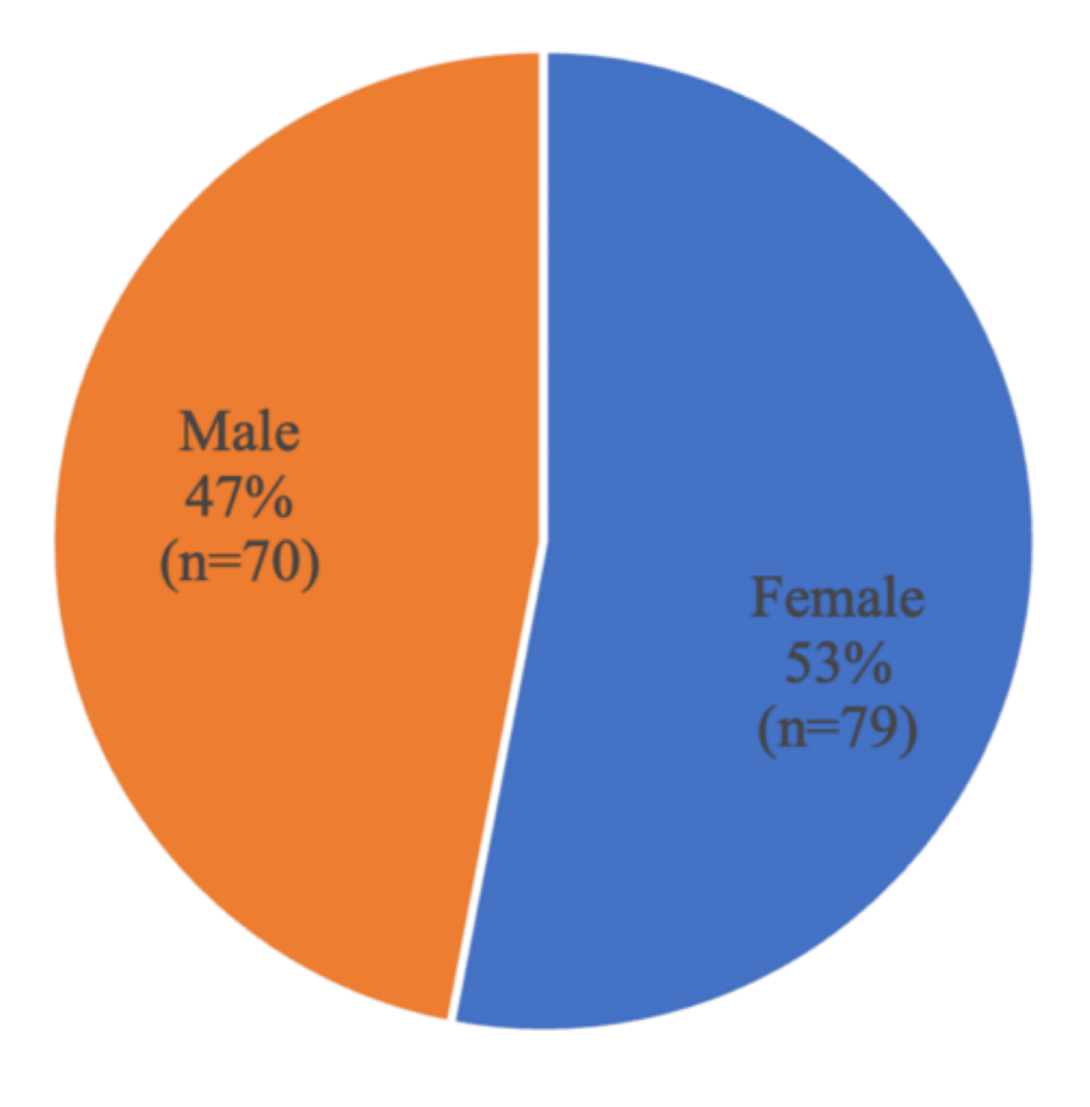
Gender distribution

**Figure 3:**
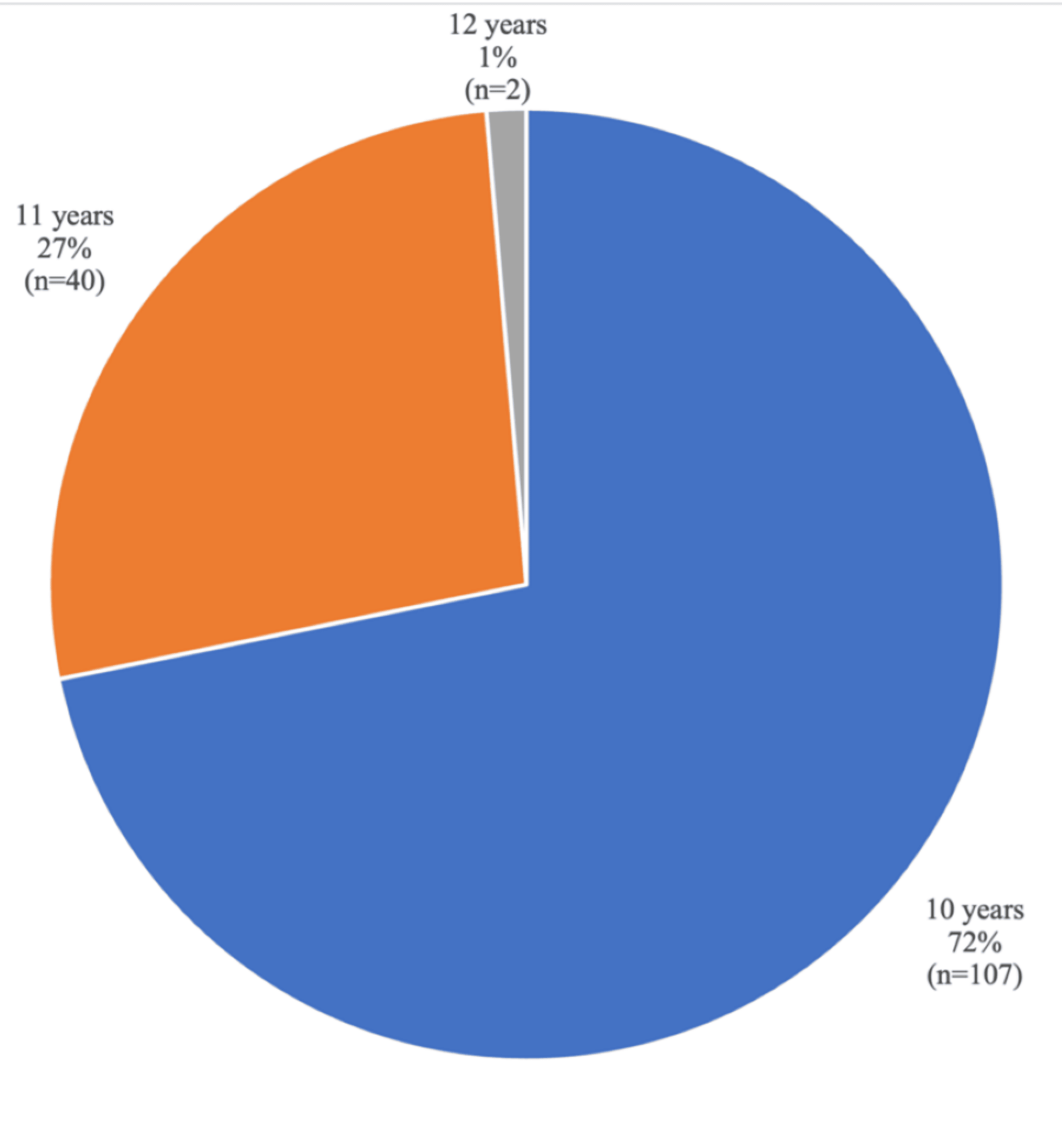
Age distribution

The study population consisted of 149 fifth-grade elementary school students. Regarding gender distribution, a slight female predominance was observed, with 53% of participants being female (Figure 2). Participant ages ranged from 10 to 12 years (Figure 3). There were no losses to follow-up: all students who attended the educational intervention completed the questionnaire at all subsequent evaluation time points.

Questionnaire results and effects of the intervention on stroke knowledge

Before the educational intervention, the mean questionnaire score was 6.1 ± 1.9 (n = 149). Immediately after the intervention, the mean score increased to 8.7 ± 1.3 (n = 149), representing a 42.6% improvement compared with baseline. One month after the intervention, the mean score slightly decreased to 8.1 ± 1.3 (n = 149), and at one year to 7.6 ± 1.8 (n = 149), corresponding to relative improvements of 32.8% and 24.5%, respectively, compared with baseline. 

**Figure 4:**
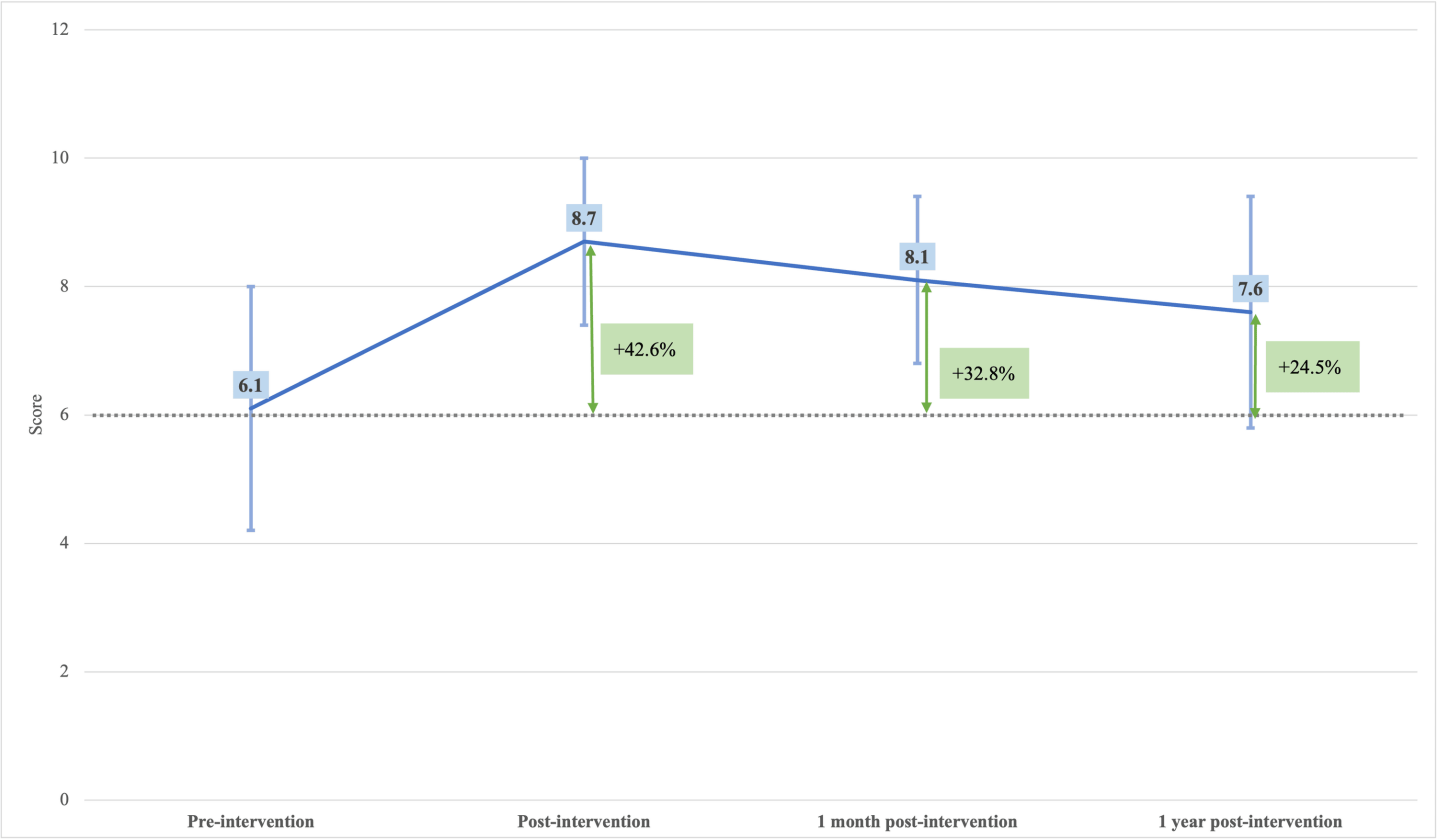
Mean questionnaire scores at each evaluation period, with the percentage improvement relative to the pre-intervention baseline (p < 0.0001, one-way ANOVA)

To assess changes in stroke-related knowledge over time, a one-way ANOVA was performed to compare mean questionnaire scores (range: 0-10 points) across the four evaluation periods: pre-intervention, immediately post-intervention, one month post-intervention, and one year post-intervention. The analysis demonstrated a statistically significant effect of time (p < 0.0001), indicating that the educational intervention was associated with significant improvements in students’ knowledge over the study period (Figure 4).

Bonferroni-adjusted post-hoc comparisons showed that mean scores increased significantly from pre-intervention to immediately post-intervention (p < 0.001), one month post-intervention (p < 0.001), and one year post-intervention (p < 0.001). Scores immediately after the intervention were also significantly higher than those at one month (p < 0.001), and one year (p < 0.001). No statistically significant difference was observed between the one-month and one-year assessments after Bonferroni correction (p = 0.011, not significant) (Table 1).

**Table 1:** Pairwise comparisons of questionnaire scores across time points, with Bonferroni-corrected p-values

Comparison	t-test p-value (Bonferroni-corrected)	Significant
Pre vs. post-intervention	p < 0.001	Yes
Pre vs. 1 month post-intervention	p < 0.001	Yes
Pre vs. 1 year post-intervention	p < 0.001	Yes
Post vs. 1 month post-intervention	p < 0.001	Yes
Post vs. 1 year post-intervention	p < 0.001	Yes
1 month vs. 1 year post-intervention	p = 0.012	No

Independent-samples t-tests were used to compare questionnaire scores between male and female students at each evaluation point. Although t-tests assume an approximately normal distribution, they are considered robust to deviations from normality in large samples such as this one (n = 149). As Microsoft Excel® does not support non-parametric alternatives, such as the Mann-Whitney U test, this limitation was acknowledged, and gender comparisons were interpreted with appropriate caution. No statistically significant differences were observed between male and female students at any time point: pre-intervention (p = 0.973), immediately post-intervention (p = 0.995), one month post-intervention (p = 0.763), and one year post-intervention (p = 0.714) (Table 2). Overall, stroke knowledge scores were comparable between genders throughout the study period.

**Table 2:** Comparative analysis of questionnaire results between female and male participants at each evaluation time point using independent-samples t-tests

Time point	Male, mean ± SD	Female, mean ± SD	t-test p-value	Significant
Pre-intervention	6.13 ± 1.87	6.12 ± 1.88	0.973	No
Post-intervention	8.74 ± 1.26	8.71 ± 1.25	0.995	No
1 month post-intervention	8.10 ± 1.25	8.07 ± 1.25	0.763	No
1 year post-intervention	7.62 ± 1.82	7.60 ± 1.82	0.714	No

When questionnaire items were grouped by thematic area, baseline performance varied substantially. In the single-item domains (maximum of 149 correct responses), 91 students (61%) correctly answered the stroke definition question, while 32 students (22%) correctly answered the epidemiology question. In the two-item domains (maximum of 298 correct responses), baseline correct responses were 105 (35%) for pathophysiology, 187 (63%) for symptom recognition, 250 (84%) for appropriate action in suspected stroke, and 241 (81%) for primary prevention.

Immediately after the intervention, a marked increase in correct responses was observed across all thematic areas except pathophysiology. Post-intervention correct responses increased to 137 (92%) for definition, 98 (66%) for epidemiology, 203 (68%) for pathophysiology, 289 (97%) for symptom recognition, 294 (99%) for appropriate action, and 278 (93%) for primary prevention.

At the one-month follow-up, a slight decline was observed; however, performance remained well above baseline levels, with 129 correct responses (87%) for definition, 63 (42%) for epidemiology, 158 (53%) for pathophysiology, 279 (94%) for symptom recognition, 285 (96%) for appropriate action, and 276 (93%) for primary prevention.

**Figure 5:**
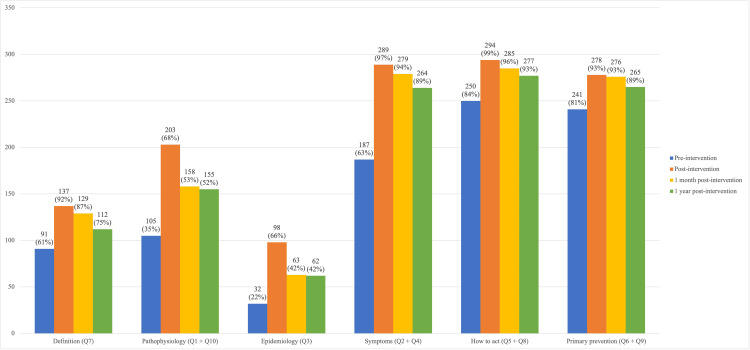
Number and percentage of correct answers by thematic area at each evaluation period For themes with one question, the maximum n = 149; for themes with two questions, the maximum n = 298.

At the one-year assessment, performance declined further but remained consistently higher than baseline across all domains. Correct responses were 112 (75%) for definition, 62 (42%) for epidemiology, 155 (52%) for pathophysiology, 264 (89%) for symptom recognition, 277 (93%) for appropriate action, and 265 (89%) for primary prevention. Overall, the strongest and most sustained gains were observed in symptom recognition, appropriate action in suspected stroke, and primary prevention, whereas greater declines were noted in definition, pathophysiology, and epidemiology (Figure 5).

## Discussion

The educational intervention implemented in this study resulted in significant improvements in students’ knowledge about stroke. As expected, the most pronounced increase in mean scores occurred immediately after the intervention, a finding consistent with previous school-based stroke education and health awareness studies, which commonly report substantial short-term knowledge gains following structured instructional sessions [15,18,21]. Although a gradual decline in knowledge retention was observed over time, all post-intervention scores remained well above baseline levels throughout the one-year follow-up, demonstrating sustained knowledge acquisition and retention [21-24].

When analysing performance by thematic area, the most stable long-term knowledge gains were observed in the recognition of stroke symptoms, appropriate actions to take when a stroke is suspected, and primary prevention measures. These findings are consistent with evidence from similar educational programmes, which indicate that children and adolescents can effectively learn and retain practical information related to emergency recognition and prevention strategies [23,25,26]. From a public health perspective, these domains are particularly relevant, as early identification of stroke warning signs and prompt activation of emergency medical services are key determinants of timely treatment and improved clinical outcomes [19,26].

In contrast, greater knowledge decay was observed in areas related to pathophysiology and epidemiology. This pattern has also been described in other educational interventions and is likely attributable to the more abstract and theoretical nature of these concepts, which are less directly applicable to children’s daily experiences [12]. Importantly, this decline does not compromise the primary aims of the intervention, which focused on improving knowledge related to symptom recognition and appropriate responses in suspected stroke situations.

Another relevant aspect of this study is that the intervention was conducted in a public school attended by students from diverse socioeconomic backgrounds. This heterogeneity reduces the likelihood that the observed results were strongly influenced by parental educational level or socioeconomic status, thereby supporting the potential applicability of similar school-based education interventions in comparable settings [15].

This study has several limitations. First, only theoretical knowledge was assessed; behavioural outcomes or real-life application of the acquired information were not evaluated. Consequently, it is not possible to determine whether increased knowledge translates into behavioural change or measurable health outcomes. Second, the study was conducted in a single school and relied on convenience sampling, which limits the generalisability of the findings. Future research would benefit from multi-school or region-wide implementations, larger and more diverse samples, and the inclusion of behavioural or outcome-based measures. Additionally, the use of Microsoft Excel® for statistical analysis limited access to non-parametric tests such as the Mann-Whitney U test. However, given the large sample size and the very small differences observed between male and female students, this limitation is unlikely to have influenced the interpretation of gender comparisons. 

An additional important finding was that most knowledge decay occurred between the immediate post-intervention assessment and the one-month follow-up, after which performance remained relatively stable up to one year. This pattern suggests that, while a single educational intervention is effective in increasing stroke-related knowledge, early reinforcement may help consolidate learning and minimise initial knowledge loss. Although the optimal frequency, format, and timing of reinforcement sessions were not examined in this study, these findings support the potential value of periodic refresher activities to enhance long-term retention, as suggested by previous research [15,21,24,26].

Finally, addressing multiple thematic domains - including symptom recognition, emergency response, prevention, epidemiology, and basic mechanisms - allowed for a comprehensive educational approach focused on stroke awareness and understanding. This reinforces the value of integrating structured, age-appropriate, and interactive educational strategies into school health programmes as a means of strengthening stroke-related knowledge among children [12-14].

## Conclusions

The Projeto Alerta AVC (Stroke Alert Project) educational intervention significantly improved stroke-related knowledge among fifth-grade school students, particularly regarding recognition of warning signs, appropriate emergency actions, and primary prevention measures. Although some decline in knowledge was observed over time, scores at one month and one year remained consistently above baseline, indicating sustained knowledge retention.

These findings support the integration of structured, age-appropriate stroke education in school health programmes. Expanding similar interventions to additional schools may enhance stroke awareness and promote timely activation of emergency services; however, future research is needed to determine whether gains in knowledge translate into behavioural change and measurable health outcomes.

## Appendices

Appendix A: Stroke alert - questionnaire

Age:

Gender: Female ______ Male _____

Answer the following questions by circling the correct option:

1. Stroke:

a) Happens when the brain receives more blood flow.

b) Can lead to the death of brain cells.

c) Happens when the brain receives more nutrients.

d) Can lead to the death of heart cells.

2. What symptoms can help to recognise a stroke?

a) Sudden numbness on one side of the body, trouble speaking, loss of balance.

b) Fever, sore throat, coughing.

c) Stomach pain, nausea, diarrhea.

d) Itchy eyes, sneezing, runny nose.

3. Tick the False statement:

a) Stroke is the main cause of death in Portugal.

b) Every second someone in the world has a stroke.

c) Every 15 seconds someone in the world dies from a stroke.

d) Time is brain. Many survivors live with permanent disabilities.

4. In which situation should you suspect a stroke?

a) Grandpa complains of a severe gastric pain.

b) Grandpa complains of severe chest pain.

c) Grandpa’s speech is odd, slurring his words and you can’t understand what he’s saying.

d) Grandpa complains of severe back pain.

5. If you are next to someone who might be having a stroke, what should you do?

a) Ignore it and wait for an adult to arrive.

b) Offer water or food.

c) Call your friends.

d) Find an adult and ask them to call emergency services.

6. How can a stroke be prevented?

a) Eating pizza and French fries.

b) Spending a lot of time watching TV or playing computer games.

c) Having a healthy diet, eating fruits and vegetables every day.

d) Drinking soda or sugary drinks with meals.

7. What does “AVC” (Stroke) mean?

a) Cardiac Vascular Attack.

b) Heart Attack.

c) Cerebrovascular Accident.

d) Thrombosis.

8. What should you do if someone shows changes in speech, strength, or face?

a) Look for an adult and dial emergency services.

b) Panic and cry.

c) Calm the person down and wait for symptoms to improve.

d) Ignore the situation.

9. How can we avoid having a stroke?

a) Sitting for long periods, eating whatever we want, smoking and drinking beer.

b) Eating a healthy diet, exercising, maintaining a healthy weight and avoiding smoking and alcohol.

c) Stroke only happens to older people, so I don’t need to worry about it yet.

d) Controlling blood pressure and blood sugar is not important for stroke prevention.

10. Why does a stroke occur?

a) There is an interruption of blood flow to a certain area of the brain; as this area stops receiving oxygen and nutrients, it begins to malfunction, causing changes in face, strength, and speech.

b) There is an interruption of blood flow to a certain area of the heart; this area stops receiving the necessary blood and nutrients and symptoms such as chest pain and shortness of breath appear.

c) There is an interruption of blood flow to the leg and face, causing weakness and changes in those areas, as well as speech difficulties.

d) It happens because of excessive alcohol and tobacco use, which cause changes in strength, speech and facial movement.
